# Supplementary material for: Home Cooking Is Related to Potential Reduction in Cardiovascular Disease Risk among Adolescents: Results from the A-CHILD Study
Source: Nutrients. 2020 Dec 16;12(12):3845. doi: 10.3390/nu12123845 (PMC7766427; doi:10.3390/nu12123845)
Supplement: Supplementary file 1 [file nutrients-12-03845-s001.pdf]

Table 1. Mediation analyses between low frequency of home cooking and adolescents' indicators of cardiometabolic factors among Japanese junior high school children.

Mediator	DBP		HDL	
	**Proportion Mediated, %**	***p* Value for Estimated Indirect Effect**	**Proportion Mediated, %**	***p* Value for Estimated Indirect Effect**
Adolescent’s BMI	2.2	0.58	6.3	0.56
Adolescent’s frequency of vegetable intake	−3.4	0.55	9.7	0.09
Adolescent’s frequency of breakfast consumption	12.3	0.19	14.9	0.10

BMI: body mass index. Frequency of home cooking was treated as a binary, with 0 = middle-high frequency of home cooking and 1 = low frequency of home cooking. Models: Adjusted for child's sex, household income and mother's diabetes.
